# Edaravone's Safety Profile in Acute Ischemic Stroke

**DOI:** 10.1002/brb3.70158

**Published:** 2024-12-16

**Authors:** Laurence K. J. Batino, Cyrus G. Escabillas, Jose C. Navarro

**Affiliations:** ^1^ Department of Neurology, Zeenat Qureshi Stroke Institute Jose R. Reyes Memorial Medical Center Manila Philippines

**Keywords:** acute ischemic stroke, antioxidant, edaravone, neuroprotective agent

## Abstract

**Background:**

We aimed to evaluate the safety of intravenous edaravone for the treatment of acute ischemic stroke among Filipino patients. The study, categorized as Phase IV, spans from December 2022 to November 2023. The primary objective is to document side effects and serious adverse events during the 14‐day edaravone infusion period.

**Methods:**

The protocol gained approval from the Institutional Review Board, and participants provided written consent. Inclusion criteria involved patients aged 18–70 with acute ischemic stroke within 24 h. Exclusion criteria included extremes of age, pregnancy, severe hepatic impairment, and participation in other clinical trials. Edaravone was administered for 14 days, underwent continuous monitoring, and adverse events were actively recorded.

**Findings:**

Out of 64 enrolled patients, 58 completed the treatment, while 4 did not finish, and 2 dropped out. The majority were male (*n* = 35), median age of 53.5 years, and 81.03% exhibited moderate stroke severity. Two patients reported headaches, and one reported dizziness. No serious adverse events or other untoward effects were documented. Dropouts, attributed to a low ejection fraction, showed normal laboratory results and no side effects during edaravone infusion. Thrombolytic therapy was given to 37.93% of patients.

**Discussion:**

Our study contributes insights into edaravone's safety, revealing a favorable profile with mild side effects, aligning with existing literature. Notably, no serious adverse events occurred, emphasizing edaravone's tolerability. Headache and dizziness, which were the common side effects in our case, did not lead to treatment discontinuation. The findings support the growing evidence of edaravone's safety in acute ischemic stroke treatment. Overall, edaravone demonstrates promise in stroke management, necessitating vigilant monitoring, especially considering individual cardiovascular health.

## Graphical abstract

Participants: 64 enrolled, 58 completed edaravone treatment, 4 did not finish, 2 dropped out.
 Demographics: Majority male (*n* = 35), median age 53.5 years, 81.03% with moderate stroke severity.
 Findings: Two patients reported headaches, one reported dizziness; no serious adverse events documented.
 Dropouts: Attributed to low ejection fraction; normal lab results and no side effects during infusion.
 Thrombolytic Therapy: Administered to 37.93% of patients.

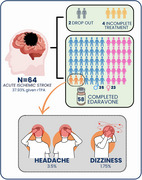

## 1 Introduction

Focal cerebral ischemia following stroke results in loss of function because of the interruption of cerebral blood supply (Go et al. 2014). It accounts for 85% of all types of stroke and is the leading cause of death and acquired disability worldwide (World Health Organization n.d.). The global burden of the disease is expected to rise greatly in the succeeding decade (Navarro et al. 2014). The socioeconomic point of view of stroke in low‐income countries has increased in age 65 and younger. This may be due to the fact that high‐income countries have good health systems, comprehensive stroke prevention and care, and numerous resources and manpower (Navarro et al. 2014).

Scientific research has significantly advanced our understanding of the mechanisms of tissue injury following cerebral ischemia (National Institute of Neurological Disorders and Stroke rt‐PA Stroke Study Group 1995). However, the current standard treatment for acute stroke, intravenous thrombolysis, is only effective within a specific therapeutic window. In developing countries like the Philippines, the use of thrombolysis remains low compared to neighboring nations, emphasizing the need for additional therapeutic options to address the debilitating effects of stroke (Heiss et al. 1998). During ischemia, several metabolic processes are accelerated, including free radical formation, increased arachidonic acid pathway products, neurotransmitter depletion, and endoplasmic reticulum dysfunction (Demchuk, Felberg, and Alexandrov 1999; Xu, Wang, and Wang 2013). These sequential events of the ischemic cascade can last for hours or days, even after the blood supply is restored. Pharmacological neuroprotection in acute stroke management aims to counteract these biochemical effects, preventing further brain tissue damage and improving clinical outcomes.

Edaravone, an oxygen radical scavenger, was first studied in Japan to treat acute ischemic stroke (Pandian and Sudhan 2013). The formation of free radicals in ischemic stroke plays an important role. The inability of the ischemic tissue to defend itself against oxidative stress leads to drastic tissue damage via several cellular pathways, leading to cell death or necrosis (Minematsu et al. 2012). Therefore, the free radicals are the main target of edaravone, which ameliorates the damaging effect of free radicals by preventing cerebral edema, further cerebral infarction and inhibits delayed neuronal death (Yamaguchi, Kikuchi, and Hayakawa 1995). In both human and animal models, edaravone inhibits peroxidation and protects neuronal populations as well as endothelial cells (Yamaguchi et al. 2006).

Edaravone, widely used for acute ischemic stroke in many countries, has demonstrated efficacy in both clinical and experimental studies since 2001. It has also been studied in other conditions involving free radical production, such as intracerebral hemorrhage (Ramaiah and Yan 2013), hypertension (Santos 2016), atherosclerosis (Kongbunkiat et al. 2016), diabetes (Navarro 2005), myocardial infarction (Tsujita et al. 2004), and heart failure (Higashi et al. 2006). When combined with recombinant tissue plasminogen activator (Alteplase), edaravone enhanced recanalization rates, with a significant improvement in recovery as measured by the NIHSS (Yagi et al. 2009). In Japan, combination therapy with antiplatelets and edaravone was incorporated into acute stroke management guidelines in 2009 (Kern et al. 2013). The EDO trial further explored the effects of combining edaravone with ozagrel. The Edaravone–Citicoline Comparative Study in Acute Ischemic Stroke (ECCS‐AIS) (Mitta et al. 2012) evaluated the efficacy of edaravone and citicoline alongside standard stroke treatment. After 3 months, the edaravone group showed significantly better neurological outcomes, with the lowest NIHSS scores (*p* = 0.000), indicating superior effectiveness compared to the citicoline group. Sub‐analysis also confirmed better outcomes for the edaravone group across different stroke severity levels (Mitta et al. 2012).

### 1.1 Rationale

The potential benefits of edaravone in clinical practice are promising. However, in the Philippines, the utilization of this drug has not been explored and postmarketing surveillance is needed to provide data regarding its response and side effects compared to other diverse populations. Safety surveillance is essential in choosing the proper medication for our patients. To address this, it is important to provide recommendations for the evaluation of the safety of edaravone among Filipinos with acute ischemic stroke. Edaravone has been proven to be safe for stroke, where only diarrhea and nausea are the most commonly reported adverse effects (Feng et al. 2011). Most side effects of edaravone recovered with treatment (Edaravone Product Insert n.d.; Otomo 2003).

In this prospective study, we evaluated and investigated the safety of edaravone treatment in Filipino patients admitted for acute stroke in a tertiary government hospital. Several studies cited above have shown that edaravone is effective as a neuroprotective agent in inhibiting the effects of acute ischemic stroke. The information generated in this study will contribute to the improvement and upgrade of clinical practice guidelines for the management and care of acute stroke victims in the Philippines.

## 2 Methods

### 2.1 Protocol Design

The protocol underwent thorough scrutiny and gained approval from the Institutional Review Board and Ethics Committee of Jose R. Reyes Memorial Medical Center. Before participation, all individuals provided written consent. This study, categorized as Phase IV, is a single‐center, open‐label, single‐arm, prospective, longitudinal investigation assessing the safety of edaravone in the treatment of acute ischemic stroke among Filipino patients. The study encompasses from December 2022 to November 2023. The primary objective of the study was to document side effects and serious adverse events, with continuous monitoring conducted throughout the 14‐day edaravone infusion period.

### 2.2 Participant Criteria

Inclusion criteria for patients were as follows: (1) Age between 18 and 70 years, experiencing acute ischemic stroke within 24 h as confirmed by CT scan; (2) measurable focal deficit on the NIH Stroke Scale; (3) level of consciousness between 0 (alert) and 3 (unable to recall name and date of birth) according to the Japan Coma Scale; 4) Motor dysfunction in upper and/or lower extremities. Exclusion criteria comprised: individuals below 18 or above 70 years of age; pregnant and lactating patients; those with an unestablished time of stroke onset; individuals refusing to provide consent; patients with severe hepatic impairment or serum creatinine levels exceeding 1.5 mg/dL; those with intracranial hemorrhage or transient ischemic attack; individuals receiving specific drug treatments after stroke onset; those undergoing surgical or intravascular treatments; patients with severe complications (e.g., cirrhosis, heart failure); individuals undergoing treatment for malignant tumors; and those participating in other clinical trials or postmarketing studies within the last 3 months.

### 2.3 Screening and Treatment

Participants underwent an assessment to determine their adherence to inclusion and exclusion criteria. The Informed Consent Process was conducted by the investigators, elucidating the study's purpose, procedures, and participant rights, ensuring comprehensive understanding before participants, or their designated representative, signed the written Informed Consent Form (ICF). A thorough medical history was obtained, and a comprehensive physical and neurologic examination, including vital sign measurements, was conducted.

Upon ICF completion, the subsequent information and screening procedures were obtained: baseline demographics: age, sex, risk factors, and comorbidities. Screening laboratory and ancillary tests, which included complete blood count, encompassing hemoglobin, hematocrit, total white cell count, differential count, and platelet count, cranial CT scan, liver function test: alanine aminotransferase (ALT) and aspartate aminotransferase (AST), serum sodium, potassium, BUN, and creatinine.

Before the infusion of edaravone, the standard of care, following clinical practice guidelines for stroke care and management, was administered. Edaravone was given intravenously as a 30‐mg dose in normal saline twice daily for 14 days, underwent immediate observation for tolerability and adverse reactions. Periodic monitoring for adverse events, serious adverse events, and suspected unexpected serious adverse reactions occurred throughout the 14 days of edaravone treatment and recorded in the Adverse Event Report Form. At the end of the study, after giving the last dose on the 14th day, the following blood tests were conducted: complete blood count, encompassing hemoglobin, hematocrit, total white cell count, differential count, and platelet count, liver function test: ALT and AST, serum sodium, potassium, BUN, and creatinine.

### 2.4 Side Effect and Adverse Reaction Observation

The adverse event was defined as “any untoward medical occurrence following drug administration, not necessarily causally linked to the drug's use.” It encompassed any unfavorable or unintended sign, abnormal laboratory finding, symptom, or disease. Active monitoring of adverse events occurred throughout the 14‐day edaravone treatment. Trained healthcare professionals conducted active surveillance and reporting, documenting all adverse events on a designated form during the entire 14‐day treatment period. Throughout active surveillance, information concerning participants' overall condition was gathered.

In addition to actively following up on adverse events noted during the drug administration period, any other adverse or serious adverse events occurring from the study's initiation to its conclusion were reported in the Case Report Form.

### 2.5 Statistical Analysis

This study assessed the safety of edaravone in treating acute stroke among Filipino patients.

The sample size calculation drew from a previous study on edaravone, examining its safety and efficacy profile. In that study, potential adverse effects showed a 16% incidence in a sample of 194 subjects (Witzel et al. 2022). Expressing a single‐arm study with a sample size of *N* participants in terms of the probability of concluding the regimen's acceptability, with 90% power, a 97% confidence interval, a margin of error (0.5), and 5% significance, the computed sample size required was 64. The equation used for this calculation is as follows (OpenEpi version 3, open‐source calculator):

$$\begin{array}{ccc} \text{Sample size}\;n & = & [\mathrm{DEFF}\times Np\left(1-p\right)l\\ & & /\left(a2/23-c/2\times \left(N-1\right)+p\times \left(1-p\right)\right)] \end{array}$$

### 2.6 Safety Analysis

Descriptive statistics were employed for the analysis of adverse events. The incidence of adverse events and adverse drug reactions was calculated as the ratio of patients who experienced specific adverse events to the total number of patients. The temporal relationship regarding the existing data of the side effects was compared with our data.

## 3 Results

A total of 64 patients were initially enrolled in the study, with 58 successfully completing the 14‐day edaravone treatment. Four patients could not complete the study due to scarcity, and two others dropped out. Table 1 presents a comprehensive overview of the clinical and demographic characteristics of the 58 patients who completed the treatment. The majority were male (*n* = 35), with a median age of 53.5 years (ranging from 37 to 70 years). NIHSS scores varied from 5 to 22, with 81.03% of patients showing moderate stroke severity. In addition, 22 patients (37.93%) received rtPA treatment. Common comorbidities and risk factors included hypertension (47 patients), diabetes (12 patients), atrial fibrillation (2 patients), gouty arthritis (1 patient), smoking history (16 patients), alcohol consumption (9 patients), drug use (4 patients), and chronic oral contraceptive pill use (1 patient).

**TABLE 1:** Clinico‐demographic profiles of acute ischemic stroke patients who were given edaravone for 14 days (*n *= 58).

Clinico‐demographic variables	Frequency	Percentage
Age		
Young adulthood (20–39 years old)	2	3.44%
Middle adulthood (40–59 years old)	36	62.06%
Old age (above 59 years old)	20	34.48%
Gender		
Male	35	60.34%
Female	23	39.66 %
Given rTPA	22	37.93%
NIHSS upon admission		
5	10	17.24%
6–21	47	81.03%
22 or more	1	1.72%
NIHSS on discharge		
Minor stroke (1–4 points)	45	77.58%
Moderate stroke (5–15 points)	12	20.69%
Moderate/severe stroke (16–20 points)	0	0%
Severe stroke (more than 20 points)	0	0%
Comorbidities		
None	2	3.44%
Hypertension	47	81.03%
Known atrial fibrillation	2	3.44%
Dyslipidemia	43	74.14
Diabetes	12	20.69%
Gouty arthritis	1	1.72%
Obstructive sleep apnea	0	0
Other risk factors		
Smoker	16	27.58%
Alcoholic	9	15.52%
Drug user	4	6.90%
OCP user	1	1.72%

Laboratory results for all patients, including complete blood count, platelet count, creatinine, blood urea nitrogen, ALT, AST, serum sodium, and potassium, were normal both before and after the edaravone infusion. This indicates that the treatment did not lead to any significant biochemical abnormalities. During the 14‐day infusion period, two patients reported headaches and one experienced dizziness. Notably, no other adverse reactions such as dyspnea, chest pain, palpitations, nausea, vomiting, or diarrhea were observed, suggesting that edaravone was generally well‐tolerated.

Table 2 details the clinical and demographic characteristics of the four patients who did not complete the treatment. These individuals also exhibited normal laboratory results before and after edaravone administration, and there were no recorded side effects or serious adverse events during the infusion.

**TABLE 2:** Clinico‐demographic profiles and duration of edaravone of acute ischemic stroke patients who did not complete edaravone (*n* = 4).

Clinico‐demographic variables	Frequency	Percentage
Age		
Young adulthood (20–39 years old)	0	0%
Middle adulthood (40–59 years old)	4	100%
Old age (above 59 years old)	0	0%
Gender		
Male	2	50%
Female	2	50%
Given rTPA	0	0%
NIHSS upon admission		
5	1	25%
6–21	3	75%
22 or more		0%
NIHSS on discharge		
Minor stroke (1–4 points)	3	75%
Moderate stroke (5–15 points)	1	25%
Moderate/severe stroke (16–20 points)	0	0%
Severe stroke (more than 20 points)	0	0%
Comorbidities		
None	0	0%
Hypertension	3	75%
Known atrial fibrillation	2	50%
Dyslipidemia	3	75%
Diabetes	1	25%
Gouty arthritis	0	0%
Obstructive sleep apnea	0	0%
Other risk factors		
Smoker	2	50%
Alcoholic	1	25%
Drug user	0	0%
OCP user	0	0%
Number of days edaravone given		
4 days	1	25%
8 days	1	25%
10 days	1	25%
12 days	1	25%

The two dropouts were due to a low ejection fraction discovered on the eighth day of edaravone infusion during routine 2D echocardiography. Although these patients had normal laboratory results and no reported side effects during the treatment, edaravone administration was discontinued as a precautionary measure due to the identified low ejection fraction. The causality between edaravone exposure and the low ejection fraction is unclear, as there were no direct symptoms or lab abnormalities linked to the treatment.

Among the 58 patients who completed the treatment, 3 experienced mild side effects, as summarized in Table 3. Two patients reported headaches, and one complained of dizziness. These symptoms were not severe enough to warrant discontinuation of the treatment, indicating a low incidence of mild adverse events and no serious complications directly attributable to edaravone. The absence of significant biochemical changes and severe side effects reinforces the safety profile of edaravone in treating acute ischemic stroke.

**TABLE 3:** Side effects (*n* = 3).

Side effects	Side effect details	Age/sex	Infarct	Risk factors	Given rTPA	Baseline NIHSS	NIHSS on discharge
Headache (3.5%)	Left parietal headache, intermittent 5–7/10 on pain analog scale throbbing headache (relieved with Paracetamol IV)	45/M	Right striatocapsular infarct	Hypertensive, diabetic, smoker, drug user	No	**15**	**3**
	Left frontal headache, intermittent, 3–5/10 on pain analog scale (relieved with Paracetamol IV)	70/M	Partial anterior circulation infarction, LMCA territory	Hypertensive, smoker, alcoholic	Given	**7**	**0**
Dizziness (1.75%)	Relieved with cinnarizine	50/M	Lacunar infarct	Hypertensive, diabetic	Given	**6**	**0**

## 4 Discussion

Our study evaluated the safety profile of edaravone during a 14‐day treatment period for acute ischemic stroke among Filipino patients. The primary findings demonstrate a highly favorable safety profile, with 58 of 64 patients completing the treatment without experiencing severe adverse effects. Notably, no cases of skin rash, pruritus, dyspnea, chest pain, palpitation, nausea, vomiting, or diarrhea were observed during the infusion period, underscoring the good tolerability of edaravone in this patient population. These results corroborate previous research emphasizing edaravone's safety and its well‐tolerated nature in the context of acute stroke management (Badillo and Navarro 2023; Chen et al. 2021; Edaravone Product Insert n.d.; Feng et al. 2011; Mitta et al. 2012; Otomo 2003).

In our cohort, headache and dizziness were the most frequently reported side effects, consistent with the literature that identifies these as common, mild adverse events associated with edaravone treatment (Witzel et al. 2022). Importantly, these side effects did not result in discontinuation of treatment for most patients, suggesting that edaravone is generally well‐tolerated by the majority of users. This finding is aligned with established data on the safety profile of edaravone, which typically highlights these mild adverse effects without leading to severe complications (Badillo and Navarro 2023; Witzel et al. 2022).

Interestingly, among the four patients who did not complete the study, there were no recorded side effects or serious adverse events linked to edaravone administration. The two patients who discontinued treatment due to a low ejection fraction, discovered on Day 8 of the treatment, had normal laboratory results and did not report any symptoms or adverse effects attributable to edaravone. This situation highlights the cautious approach taken when cardiovascular parameters are compromised, even in the absence of direct evidence connecting edaravone to the observed low ejection fraction.

A systematic review of edaravone's safety found that headache was the most commonly documented reaction, occurring in 32% of cases compared to controls (Badillo and Navarro 2023). In our study, headache was reported in 3.5% of patients, which is lower than the systematic review findings but still within the expected range for such adverse events. Dizziness was noted in 1.75% of our patients, compared to 4.62% reported by Badillo et al. (Witzel et al. 2022). These findings reinforce the importance of monitoring for potential adverse events associated with edaravone administration while affirming the drug's generally favorable safety profile.

The importance of our study lies not only in contributing to the existing body of literature but also in providing region‐specific data for the Filipino population, which may exhibit unique demographic and genetic characteristics influencing drug tolerance and safety profiles. While the safety of edaravone is well‐documented, our research offers additional validation of its tolerability in a specific patient cohort, enhancing the generalizability of the findings across diverse populations. By confirming the safety profile of edaravone in this context, our study supports its continued use and helps inform clinical decisions, ensuring that treatment recommendations are based on comprehensive and inclusive evidence.

In conclusion, our findings reinforce the safety of incorporating edaravone into standard treatment protocols for acute ischemic stroke and suggest a potential reduction in mortality risk as substantiated by various systematic reviews and meta‐analyses (Badillo and Navarro 2023; Chen et al. 2021; Fidalgo et al. 2022). Our research adds to the growing evidence favoring edaravone's use, demonstrating that the side effects are tolerable. Nonetheless, this Phase IV study is crucial as it provides real‐world evidence on the long‐term safety and efficacy of edaravone in diverse populations, such as Filipino patients with acute ischemic stroke.

## Author Contributions

**Laurence K. J. Batino**: conceptualization, methodology, data collection and analysis, review and editing. **Cyrus G. Escabillas**: conceptualization, writing–original draft. **Jose C. Navarro**: conceptualization, supervision, validation.

## Ethics Statement

The study was approved (2022‐087) by the Institutional Review Board and Ethics Committee of Jose R. Reyes Memorial Medical Center.

## Consent

Informed consent was obtained from all subjects or their legally‐acceptable representative before recruitment.

## Conflicts of Interest

The authors declare no conflicts of interest.

### Peer Review

The peer review history for this article is available at https://publons.com/publon/10.1002/brb3.70158.

## Data Availability

All data generated or analyzed during this study are included in this article. Further inquiries can be directed to the corresponding author.

## References

[brb370158-bib-0001] Badillo, S. , and J. Navarro . 2023. “Safety of Edaravone in Acute Ischemic Stroke: A Systematic Review and Meta‐Analysis.” Neurology Asia 28, no. 1: 39–53.

[brb370158-bib-0002] Chen, C. , M. Li , L. Lin , S. Chen , Y. Chen , and L. Hong . 2021. “Clinical Effects and Safety of Edaravone in Treatment of Acute Ischaemic Stroke: A Meta‐Analysis of Randomized Controlled Trials.” Journal of Clinical Pharmacy and Therapeutics 46, no. 4: 907–917. 33638896 10.1111/jcpt.13392 PMC8359409

[brb370158-bib-0003] Demchuk, A. M. , R. A. Felberg , and A. V. Alexandrov . 1999. “Clinical Recovery from Acute Ischemic Stroke After Early Reperfusion of the Brain With Intravenous Thrombolysis.” New England Journal of Medicine 340: 894–895. 10084910 10.1056/NEJM199903183401117

[brb370158-bib-0004] Edaravone Product Insert . n.d. NanJing: Simcere Dongyuan Pharmaceutical Co., Ltd. China. https://verification.fda.gov.ph/files/DR‐XY48223_PI_01.pdf.

[brb370158-bib-0005] Feng, S. , Q. Yang , M. Liu , et al. 2011. “Edaravone for Acute Ischaemic Stroke.” Cochrane Database of Systematic Reviews 12: CD007230. 10.1002/14651858.CD007230. PMC12668575 22161410

[brb370158-bib-0006] Fidalgo, M. , J. Ricardo Pires , I. Viseu , et al. 2022. “Edaravone for Acute Ischemic Stroke—Systematic Review With Meta‐Analysis.” Clinical Neurology and Neurosurgery 219: 107299. 35753163 10.1016/j.clineuro.2022.107299

[brb370158-bib-0007] Go, A. S. , D. Mozaffarian , V. L. Roger , et al. 2014. “Heart Disease and Stroke Statistics–2014 Update: A Report From the American Heart Association.” Circulation 129: e28–e292. 24352519 10.1161/01.cir.0000441139.02102.80 PMC5408159

[brb370158-bib-0008] Heiss, W.‐D. , M. Grond , A. Thiel , et al. 1998. “Tissue at Risk of Infarction Rescued by Early Reperfusion: A Positron Emission Tomography Study in Systemic Recombinant Tissue Plasminogen Activator Thrombolysis of Acute Stroke.” Journal of Cerebral Blood Flow and Metabolism 18: 1298–1307. 9850142 10.1097/00004647-199812000-00004

[brb370158-bib-0009] Higashi, Y. , D. Jitsuiki , K. Chayama , and M. Yoshizumi . 2006. “Edaravone (3‐Methyl‐1‐Phenyl‐2‐Pyrazolin‐5‐One), a Novel Free Radical Scavenger, for Treatment of Cardiovascular Diseases.” Recent Patents on Cardiovascular Drug Discovery 1, no. 1: 85–93. 18221078 10.2174/157489006775244191

[brb370158-bib-0010] Kern, R. , M. Nagayama , K. Toyoda , T. Steiner , M. Hennerici , and Y. Shinohara . 2013. “Comparison of the European and Japanese Guidelines for the Management of Ischemic Stroke.” Cerebrovascular Diseases 35, no. 5: 402–418. 23712178 10.1159/000351753

[brb370158-bib-0011] Kongbunkiat, K. N. K. , S. Tiamkao , V. Chotmongkol , et al. 2016. “Thrombolysis in Ischaemic Stroke in Rural North East Thailand by Neurologist and Non‐Neurologists.” Neurology Asia 21, no. 4: 325–331.

[brb370158-bib-0012] Minematsu, K. , K. Toyoda , T. Hirano , et al. 2012. “Guidelines for the Intravenous Application of Recombinant Tissue‐Type Plasminogen Activator (Alteplase), the Second Edition 2012: A Guideline From the Japan Stroke Society.” Journal of Stroke and Cerebrovascular Diseases 22, no. 5: 571–600. 10.1016/j.jstrokecerebrovasdis.2013.04.001 23727456

[brb370158-bib-0013] Mitta, M. , D. Goel , K. Bansal , and P. Puri . 2012. “Edaravone—Citicoline Comparative Study in Acute Ischemic Stroke (ECCS‐AIS).” Journal of the Association of Physicians of India 60: 36–38. 23767201

[brb370158-bib-0014] National Institute of Neurological Disorders and Stroke rt‐PA Stroke Study Group . 1995. “Tissue Plasminogen Activator for Acute Ischemic Stroke.” New England Journal of Medicine 333, no. 24: 1581–1588. 7477192 10.1056/NEJM199512143332401

[brb370158-bib-0015] Navarro, J. C. 2005. “Prevalence of Stroke: Community Survey.” Philippine Journal of Neurology 9: 11–15.

[brb370158-bib-0016] Navarro, J. C. , A. C. Baroque , J. K. Lokin , et al. 2014. “The Real Burden of Stroke in the Philippines.” International journal of Stroke 9, no. 5: 640–641. 24844610 10.1111/ijs.12287

[brb370158-bib-0017] Otomo, E. 2003. “Effect of a Novel Free Radical Scavenger, Edaravone (MCI‐186), on Acute Brain Infarction.” Cerebrovascular Diseases 15, no. 3: 222–229. 12715790 10.1159/000069318

[brb370158-bib-0018] Pandian, J. D. , and P. Sudhan . 2013. “Stroke Epidemiology and Stroke Care Services in India.” Journal of Stroke 15: 128–134. 24396806 10.5853/jos.2013.15.3.128 PMC3859004

[brb370158-bib-0019] Ramaiah, S. S. , and B. Yan . 2013. “Low‐Dose Tissue Plasminogen Activator and Standard‐Dose Tissue Plasminogen Activator in Acute Ischemic Stroke in Asian Populations: A Review.” Cerebrovascular Diseases 36: 161–166. 24135524 10.1159/000354162

[brb370158-bib-0020] Santos, T. G. 2016. Free Medicine Available for Stroke Victims–DOH. http://newsinfo.inquirer.net/806889/free‐medicine‐available‐for‐stroke‐victims‐doh.

[brb370158-bib-0023] Tsujita, K. , H. Shimomura , H. Kawano , et al. 2004. “Effects of Edaravone on Reperfusion Injury in Patients with Acute Myocardial Infarction.” American Journal of Cardiology 94, no. 4: 481–484. 15325934 10.1016/j.amjcard.2004.05.007

[brb370158-bib-0024] Witzel, S. , A. Maier , R. Steinbach , et al. 2022. “Safety and Effectiveness of Long‐Term Intravenous Administration of Edaravone for Treatment of Patients With Amyotrophic Lateral Sclerosis.” JAMA Neurology 79, no. 2: 121–130. 35006266 10.1001/jamaneurol.2021.4893 PMC8749709

[brb370158-bib-0025] World Health Organization . n.d. WHO Health Statistics and Information Systems. http://www.who.int/healthinfo/global_burden_disease/en/.

[brb370158-bib-0026] Xu, A. D. , Y. J. Wang , and D. Z. Wang . 2013. “Consensus Statement on the Use of Intravenous Recombinant Tissue Plasminogen Activator to Treat Acute Ischemic Stroke by the Chinese Stroke Therapy Expert Panel.” CNS Neuroscience & Therapeutics 19: 543–548. 23710819 10.1111/cns.12126 PMC6493470

[brb370158-bib-0027] Yagi, K. , K. Kitazato , M. Uno , et al. 2009. “Edaravone, a Free Radical Scavenger, Inhibits MMP‐9–Related Brain Hemorrhage in Rats Treated With Tissue Plasminogen Activator.” Stroke 40, no. 2: 626–631. 19095969 10.1161/STROKEAHA.108.520262

[brb370158-bib-0028] Yamaguchi, T. , H. Kikuchi , T. Hayakawa , et al. 1995. “Clinical Efficacy and Safety of Intravenous Tissue Plasminogen Activator in Acute Embolic Stroke: A Randomized, Double‐Blind, Dose‐Comparison Study of Duteplase.” In *Proceedings of* Thrombolytic Therapy in Acute Ischemic Stroke III, edited by T. Yamaguchi , E. Mori , K. Minematsu , and G. J. del Zoppo , 223–229. Tokyo, Japan: Springer‐Verlag.

[brb370158-bib-0029] Yamaguchi, T. , E. Mori , K. Minematsu , et al. 2006. “Alteplase at 0.6 mg/kg for Acute Ischemic Stroke Within 3 Hours of Onset: Japan Alteplase Clinical Trial (J‐ACT).” Stroke 37: 1810–1815. 16763187 10.1161/01.STR.0000227191.01792.e3

